# Association of *LIN28B* with Adult Adiposity-Related Traits in Females

**DOI:** 10.1371/journal.pone.0048785

**Published:** 2012-11-13

**Authors:** Jaakko T. Leinonen, Ida Surakka, Aki S. Havulinna, Johannes Kettunen, Riitta Luoto, Veikko Salomaa, Elisabeth Widén

**Affiliations:** 1 Institute for Molecular Medicine Finland FIMM, University of Helsinki, Helsinki, Finland; 2 National Institute for Health and Welfare, Helsinki, Finland; 3 UKK Institute for Health Promotion, Tampere, Finland; Tulane School of Public Health and Tropical Medicine, United States of America

## Abstract

**Context:**

Pubertal timing is under strong genetic control and its early onset associates with several adverse health outcomes in adulthood, including obesity, type 2 diabetes and cardiovascular disease. Recent data indicate strong association between pubertal timing and genetic variants near *LIN28B*, but it is currently unknown whether the gene contributes to the association between puberty and adult disease.

**Objective:**

To elucidate the putative genetic link between early puberty and adult disease risk, we examined the association of two genetic variants near *LIN28B* with adult body size and metabolic profiles in randomly ascertained adult Finnish males and females.

**Methods:**

Two single nucleotide polymorphisms (SNPs), rs7759938, the lead SNP previously associated with pubertal timing and height, and rs314279, previously also associated with menarcheal age but only partially correlated with rs7759938 (r^2^ = 0.30), were genotyped in 26,636 study subjects participating in the Finnish population survey FINRISK. Marker associations with adult height, weight, body mass index (BMI), hip and waist circumference, blood glucose, serum insulin and lipid/lipoprotein levels were determined by linear regression analyses.

**Results:**

Both rs7759938 and rs314279 associated with adult height in both sexes (p = 2×10^−6^ and p = 0.001). Furthermore, rs314279 associated with increased weight in females (p = 0.001). Conditioned analyses including both SNPs in the regression model verified that rs314279 independently associates with adult female weight, BMI and hip circumference (p<0.005). Neither SNP associated with glucose, lipid, or lipoprotein levels.

**Conclusion:**

Genetic variants near the puberty-associated gene *LIN28B* associate with adult weight and body shape in females, suggesting that the gene may tag molecular pathways influencing adult adiposity-related traits.

## Introduction

A wealth of epidemiological data show that the overall risk for many adult-onset multi-factorial disorders may correlate with distinct growth patterns during early development [1]. Puberty represents an example of such a developmental event. The timing of puberty is strongly regulated by genes and early timing has been associated with an increased risk for various adverse health outcomes, including obesity, type 2 diabetes, cardiovascular disease and hormone dependent cancers [2]–[5]. Multiple mechanisms likely account for these correlations, but evidence exists for shared genetic pathways influencing both puberty and adult health [6].

Although the genetic framework influencing pubertal timing in the general population remains poorly understood, recent genome-wide association (GWA) studies have initiated the identification of puberty-associated genes. The effect of *LIN28B* on pubertal timing has been particularly well established [2], [7]–[11]. Whether *LIN28B,* encoding for a protein inhibiting *let-7* microRNA biosynthesis, contributes to the risk for puberty-associated disease remains unclear. Even though variants near the gene have been associated with adult height and 2D:4D digit-length ratio in humans, it has not been implicated in the recent large GWA studies of obesity-related traits or Type 2 diabetes [12]–[15]. Nonetheless, studies in mice suggest that the *LIN28*-genes can influence energy homeostasis and glucose metabolism [16], [17]. Additionally, a longitudinal study of 1,242 men and 1,209 women implies that puberty-associated variants at *LIN28B* may associate with a transient BMI-increase from adolescence to mid-adulthood [18]. Thus, it is possible that the effects of *LIN28B* on adult energy metabolism are subtle and limited to specific periods of adolescent and adult life. In fact, epidemiological studies suggest that the genetic influences on body composition in general may become less prominent with increasing age [19].

To evaluate the impact of *LIN28B* on adult adiposity-related and metabolic traits we conducted a genetic association study in a large, homogenous Finnish study population encompassing 26,636 adult subjects aged 25 to 74 years. Our specific aim was to study two partially correlated SNPs upstream of *LIN28B*, rs7759938 and rs314279, and assess their association with adult body size, lipids and glucose metabolism 1) in the study population as a whole, and 2) in specific age groups of the population. rs7759938 has previously been robustly linked with pubertal growth and maturation [2], [7]. Association with menarcheal age (age at menarche, AAM) has also been reported for rs314279 [2]. This marker is only partially correlated with rs7759938 (pair-wise r^2^ = 0.30). However, many of the cohorts participating in the large meta-analysis study of AAM did not have data for this marker. Also, conditioned analyses interrogating the presence of two independent effects influencing pubertal timing were not carried out [2]. Even though the presence of two independent effects influencing AAM has not been confirmed, we still decided to include rs314279 in our analyses to gain a broad coverage of all potentially associated variation. The large sample size in combination with extensive phenotypic characterization makes the current study particularly well suited for detecting pleiotropic effects. Moreover, in a homogenous population such as the Finnish population, a limited number of mutations may underlie both Mendelian and complex traits, thereby improving the power to detect single associated variants.

## Materials and Methods

### Ethics Statement

All participants gave their written informed consent, and the study protocols were approved by the institutional ethical review boards of the National Institute for Health and Welfare, and the Helsinki University Hospital, Helsinki, Finland.

### Study Subjects

The study subjects were participants of the Finnish National FINRISK study, which is a series of Finnish population-based surveys, performed since 1972 at five-year intervals to characterize risk factors for non-communicable disease [20]. The individual FINRISK cohorts, stratified to contain at least 200 subjects of each sex in 10-year age groups (range 25 to 74 years), represent six distinct geographical regions in Finland. Our study included 5,616 study subjects from FINRISK 1992, 6,869 from FINRISK 1997, 8,257 from FINRISK 2002 and 5,908 study subjects from FINRISK 2007 with DNA samples available.

### Data Collection

The study participants were invited to a health inspection during which height, weight, waist and hip circumferences were measured by trained nurses using standardized procedures [20]. Height and weight were self-reported only by 94 and 92 subjects, respectively. The participants were instructed to fast for four hours and blood samples were drawn for DNA extraction and the determination of blood lipid, and lipoprotein concentrations. Blood glucose and insulin were measured from participants of FINRISK 2007 during a separate 75 g oral glucose tolerance test. Descriptive cohort statistics are shown in Table S1.

The study subjects were genotyped for rs7759938 and rs314279 using a Sequenom MassARRAY genotyping platform (Sequenom Inc., San Diego, USA). Individual genotypes were automatically called with Typer 4.0 Analyzer software, whereas the genotype clusters were manually checked. The genotyping success rate was >98% for both markers, and the minor allele frequencies (MAF) in the study sample were 0.32 for rs7759938 and 0.13 for rs314279. The observed pair-wise r^2^ between the markers was 0.30. The genotype frequencies were in Hardy-Weinberg equilibrium at both loci in all participating cohorts.

### Statistical Analysis

Linear regression analyses were carried out using PLINK [21]. Sex-stratified single marker analyses were run separately for each participating cohort, assuming an additive genetic model and adjusting for age. Because the two tested markers are only partially correlated, (r^2^ = 0.3, and D’ = 1), indicating that they are separated by a mutational event and not by recombination, we additionally evaluated the independent effect at each locus by separate analyses including both SNPs in the regression model. To measure the collinearity of the two SNPs in the regression analyses, the variants’ variance inflation factor (VIF) was computed for each anthropometric trait with R (http://www.r-project.org/). The results of the individual cohorts were meta-analysed by MetABEL (http://www.genabel.org/packages/MetABEL) in R 2.12.2. Additionally, SNP by age and SNP by sex interactions were assessed for both markers separately by multiple linear regression analysis. Although multiple tests were carried out in this study, the exact number of independent tests is not easily determined since the tested response variables are partially correlated. To maintain conservative interpretation of the analysis results, we set the significance threshold to p<0.005, corresponding to Bonferroni-correction accounting for ten independent tests.

## Results

We first assessed the effect of rs7759938 and rs314279 on adult body size (Table 1). As expected, the puberty-associated marker rs7759938 was significantly associated with adult height in both sexes (p = 2×10^−6^). However, it did not associate with weight, body mass index, or waist and hip circumference. Also rs314279 showed association with adult height (p = 0.001). Moreover, the height-increasing allele C at rs314279 significantly associated with increased weight (p = 0.001), and hip circumference (p = 0.003), mostly due to associations in females (Table 1). Even though the associations were generally stronger in females, subsequent sex-interaction analyses did not reveal any statistically significant evidence for sex-specific effects (Table S2).

**Table 1:** Association of genetic variants rs7759938 (upper panel) and rs314279 (lower panel) with adult anthropometric traits.

rs7759938		ALL		MALES			FEMALES		
RESPONSE VARIABLE (MODEL)	N (M,F)	BETA (SE)	P	BETA (SE)	P		BETA (SE)	P	
Height (A)	26379 (12258, 14121)	**0.042 (0.009)**	**2**×**10** ^−**6**^	0.032 (0.013)	0.01		**0.051 (0.012)**	**0.00002**	
Height (B)	26237 (12187, 14050)	**0.038 (0.010)**	**0.0002**	0.029 (0.015)	0.06		**0.046 (0.014)**	**0.001**	
Weight (A)	26377 (12257, 14120)	0.015 (0.009)	0.09	0.016 (0.014)	0.25		0.015 (0.013)	0.22	
Weight (B)	26235 (12186, 14049)	−0.001 (0.011)	0.93	0.009 (0.016)	0.56		−0.010 (0.015)	0.52	
BMI (A)	26375 (12256, 14119)	−0.002 (0.009)	0.78	−0.001 (0.013)	0.97		−0.004 (0.012)	0.73	
BMI (B)	26233 (12185, 14048)	−0.018 (0.010)	0.10	−0.008 (0.016)	0.63		−0.026 (0.015)	0.07	
Waist (A)	26314 (12291, 14023)	0.013 (0.009)	0.13	0.023 (0.013)	0.08		0.005 (0.012)	0.67	
Waist (B)	26173 (12220, 13953)	0.007 (0.010)	0.52	0.026 (0.015)	0.09		−0.010 (0.014)	0.48	
Hip (A)	26313 (12289, 14024)	0.009 (0.009)	0.33	0.010 (0.013)	0.45		0.007 (0.012)	0.54	
Hip (B)	26172 (12218, 13954)	−0.008 (0.010)	0.46	0.001 (0.016)	0.96		−0.015 (0.014)	0.29	
WHR (A)	26307 (12286, 14021)	0.013 (0.009)	0.14	0.026 (0.012)	0.04		0.000 (0.012)	0.98	
WHR (B)	26166 (12215, 13951)	0.021 (0.010)	0.05	**0.043 (0.015)**	**0.004**		−0.000 (0.014)	0.98	
							
**rs314279**		**ALL**		**MALES**			**FEMALES**		
**RESPONSE** **VARIABLE** **(MODEL)**	**N (M,F)**	**BETA (SE)**	**P**	**BETA (SE)**		**P**	**BETA (SE)**		**P**
Height (A)	26288 (12213, 14075)	**0.039 (0.012)**	**0.001**	0.030 (0.018)		0.09	**0.047 (0.017)**		**0.005**
Height (B)	26237 (12187, 14050)	0.010 (0.015)	0.51	0.008 (0.022)		0.70	0.011 (0.020)		0.60
Weight (A)	26286 (12212, 14074)	**0.042 (0.013)**	**0.001**	0.025 (0.019)		0.20	**0.057 (0.018)**		**0.001**
Weight (B)	26235 (12186, 14049)	0.043 (0.016)	0.006	0.020 (0.023)		0.39	**0.063 (0.021)**		**0.003**
BMI (A)	26284 (12211, 14073)	0.026 (0.013)	0.04	0.011 (0.019)		0.55	0.039 (0.017)		0.02
BMI (B)	26233 (12185, 14048)	0.040 (0.015)	0.008	0.017 (0.022)		0.44	**0.059 (0.020)**		**0.004**
Waist (A)	26224 (12246, 13978)	0.022 (0.012)	0.08	0.011 (0.018)		0.55	0.031 (0.017)		0.07
Waist (B)	26173 (12220, 13953)	0.017 (0.014)	0.24	−0.007 (0.021)		0.76	0.038 (0.020)		0.06
Hip (A)	26223 (12244, 13979)	**0.038 (0.012)**	**0.003**	0.026 (0.019)		0.17	0.048 (0.017)		0.006
Hip (B)	26172 (12218, 13954)	**0.045 (0.015)**	**0.003**	0.028 (0.023)		0.21	**0.060 (0.021)**		**0.004**
WHR (A)	26217 (12241, 13976)	−0.003 (0.012)	0.80	−0.009 (0.017)		0.59	0.003 (0.017)		0.86
WHR (B)	26166 (12215, 13951)	−0.019 (0.014)	0.18	−0.042 (0.021)		0.04	0.003 (0.021)		0.89

All phenotypes were standardized prior to analyses. Two regression models were used: Model A including age as a covariate, Model B including both the alternative SNP and age as covariates. The effect allele for both rs7759938 and rs314279 is C. BMI  =  body mass index, WHR  =  waist to hip ratio.

To assess the potential independent effects at each marker, we repeated the regression analyses including both SNPs in the regression model (Table 1). The data confirmed that the two markers associate with different phenotypes. Whereas rs7759938 completely accounted for the association with height, the observed effects on female body size were attributed to rs314279. More specifically, by including both SNPs in the analysis of height the association with rs7759938 changed only a little (p = 0.0002), whereas the effect of rs314279 was completely abolished. Also, the conditioned analyses indicated an association between the height-increasing allele at rs7759938 and increased WHR in males. In contrast, including both SNPs in the regression model resulted in unchanged or further enhanced association between rs314279 and weight, BMI and hip circumference in females (p<0.005) (Table 1).

Because multiple regression analyses of correlated predictor variables may give rise to erratic individual coefficient estimates, we tested the validity of the conditioned model by computing VIFs for the two SNPs in the analyses for each anthropometric trait (Table S3). The analyses revealed only limited levels of collinearity as evidenced by VIF <5, thereby supporting the validity of the conditioned model.

We next wanted to evaluate whether the associations between the *LIN28B* markers and body size are influenced by age. Plotting height, weight and BMI against calendar age showed strong correlation between these measures and age (Figure S1). Nonetheless, multiple regression analysis failed to reveal any evidence for significant SNP by age interaction (Table S4). Re-analysis of the data by specific age bins, covering 10-year age ranges spanning from 25 to 74 years, confirmed that the association patterns were mostly similar in all age groups (Table S5).

Finally, to test whether either of the SNPs near *LIN28B* associates with adult metabolic profiles, linear regression analyses of serum lipid, lipoprotein and insulin and plasma glucose measures were performed (Table 2). On average, roughly 17,500 study subjects (66%) had lipid measurements available for analyses, whereas glucose and insulin data from an oral glucose tolerance test was available for some 4,000 subjects (15%). We observed no significant associations with lipid or lipoprotein levels. However, rs314279 associated with fasting insulin levels in males (p = 0.003). As this was the only observed association with metabolic outcomes, and because insulin data was available only for a limited number of the study subjects, follow-up studies are needed to verify the finding.

**Table 2:** Association of genetic variants rs7759938 (upper panel) and rs314279 (lower panel) with adult metabolic profiles.

rs7759938		ALL		MALES		FEMALES	
RESPONSEVARIABLE(MODEL)	N (M,F)	BETA (SE)	P	BETA (SE)	P	BETA (SE)	P
Cholesterol (A)	17622 (7974, 9648)	−0.020 (0.011)	0.06	−0.027 (0.017)	0.11	−0.016 (0.014)	0.26
Cholesterol (B)	17530 (7930, 9600)	−0.017 (0.013)	0.19	−0.014 (0.020)	0.47	−0.019 (0.017)	0.26
HDL (A)	17617 (7973, 9644)	−0.001 (0.011)	0.94	−0.017 (0.017)	0.31	0.012 (0.015)	0.41
HDL (B)	17525 (7929, 9596)	−0.007 (0.014)	0.61	−0.032 (0.020)	0.11	0.014 (0.018)	0.44
Triglycerides^#^ (A)	17624 (7975, 9649)	−0.008 (0.011)	0.48	0.003 (0.017)	0.85	−0.017 (0.015)	0.26
Triglycerides^#^ (B)	17532 (7931, 9601)	−0.009 (0.013)	0.49	0.004 (0.020)	0.86	−0.019 (0.018)	0.28
APOA1 (A)	14302 (6428, 7874)	−0.001 (0.012)	0.91	−0.010 (0.019)	0.59	0.005 (0.016)	0.76
APOA1 (B)	14227 (6391, 7836)	−0.009 (0.014)	0.54	−0.028 (0.022)	0.22	0.004 (0.018)	0.82
APOB (A)	14443 (6496, 7947)	−0.022 (0.012)	0.07	−0.032 (0.018)	0.08	−0.014 (0.016)	0.38
APOB (B)	14368 (6459, 7909)	−0.010 (0.014)	0.49	−0.013 (0.022)	0.56	−0.008 (0.019)	0.68
FP Glucose (A)	4123 (1840, 2283)	0.007 (0.023)	0.74	0.015 (0.035)	0.67	0.002 (0.030)	0.95
FP Glucose (B)	4111 (1833, 2278)	−0.007 (0.027)	0.79	0.018 (0.040)	0.65	−0.027 (0.035)	0.45
2H Glucose (A)	4042 (1828, 2214)	−0.030 (0.023)	0.19	−0.001 (0.046)	0.99	−0.049 (0.036)	0.18
2H Glucose (B)	4032 (1822, 2210)	−0.017 (0.027)	0.43	0.011 (0.030)	0.72	−0.011 (0.027)	0.68
FS Insulin^#^ (A)	4099 (1865, 2234)	−0.013 (0.024)	0.58	0.002 (0.035)	0.96	−0.025 (0.032)	0.44
FS Insulin^#^ (B)	4088 (1859, 2229)	−0.001 (0.028)	0.98	0.076 (0.041)	0.06	−0.066 (0.038)	0.08
**rs314279**		**ALL**		**MALES**		**FEMALES**	
**RESPONSE** **VARIABLE** **(MODEL)**	**N (M,F)**	**BETA (SE)**	**P**	**BETA (SE)**	**P**	**BETA (SE)**	**P**
Cholesterol (A)	17564 (7945, 9619)	−0.022 (0.015)	0.16	−0.041 (0.023)	0.08	−0.008 (0.020)	0.71
Cholesterol (B)	17530 (7930, 9600)	−0.009 (0.018)	0.63	−0.031 (0.028)	0.28	0.007 (0.024)	0.77
HDL (A)	17559 (7944, 9615)	0.010 (0.016)	0.51	0.014 (0.024)	0.55	0.007 (0.022)	0.74
HDL (B)	17525 (7929, 9596)	0.016 (0.019)	0.42	0.040 (0.029)	0.17	−0.005 (0.026)	0.85
Triglycerides^#^ (A)	17566 (7946, 9620)	−0.004 (0.016)	0.80	0.000 (0.023)	0.99	−0.007 (0.021)	0.73
Triglycerides^#^ (B)	17532 (7931, 9601)	0.004 (0.019)	0.85	−0.003 (0.028)	0.92	0.009 (0.025)	0.73
APOA1 (A)	14258 (6403, 7874)	0.012 (0.017)	0.47	0.023 (0.027)	0.40	0.005 (0.023)	0.82
APOA1 (B)	14227 (6391, 7836)	0.019 (0.020)	0.36	0.044 (0.032)	0.17	0.001 (0.027)	0.98
APOB (A)	14400 (6472, 7928)	−0.038 (0.017)	0.02	−0.060 (0.026)	0.02	−0.021 (0.023)	0.34
APOB (B)	14368 (6459, 7909)	−0.030 (0.020)	0.14	−0.052 (0.031)	0.09	−0.013 (0.027)	0.63
FP Glucose (A)	4121 (1837, 2284)	0.036 (0.032)	0.27	0.003 (0.050)	0.95	0.059 (0.042)	0.16
FP Glucose (B)	4111 (1833, 2278)	0.041 (0.029)	0.16	−0.010 (0.058)	0.87	0.087 (0.050)	0.08
2H Glucose (A)	4042 (1826, 2216)	−0.044 (0.032)	0.17	−0.051 (0.048)	0.29	−0.038 (0.042)	0.37
2H Glucose (B)	4032 (1822, 2210)	−0.028 (0.037)	0.56	−0.065 (0.056)	0.25	−0.002 (0.050)	0.97
FS Insulin^#^ (A)	4098 (1863, 2235)	−0.031 (0.034)	0.36	−**0.152 (0.051)**	**0.003**	0.064 (0.045)	0.15
FS Insulin^#^ (B)	4088 (1859, 2229)	−0.029 (0.040)	0.46	−**0.211 (0.059)**	**0.0004**	0.118 (0.053)	0.03

All phenotypes were standardized prior to analyses. Two regression models were used: Model A including age as a covariate, and Model B including both the alternative SNP and age as covariates. Subjects using lipid lowering drugs or with lipid levels +/−5 standard deviations (SD) from the mean were excluded from the lipid/lipoprotein analysis. Only subjects with normal glucose tolerance not receiving treatment for diabetes were included in the regression analyses of glucose and serum insulin. The effect allele for both rs7759938 and rs314279 is C. ApoA1 =  Apolipoprotein A1, ApoB  =  Apolipoprotein B, FP  =  Fasting plasma. FS  =  Fasting serum, 2H glucose  =  Plasma glucose concentrations at 2 h after a 75 g oral glucose load. Participants of the oral glucose test were instructed to fast for 10 hours. ^#^ The variable was logarithm-transformed prior to the analysis.

## Discussion

In recent years, the evolutionarily conserved gene *LIN28B* has been convincingly associated with both timing of puberty and growth in height in humans [2], [7]–[12]. Highlighting its pleiotropic effects, the locus has also been associated with finger-length ratio [13]. Mice over-expressing the homologous gene, *LIN28A*, recapitulate the same phenotypes as observed in human GWA-studies, including increased growth in length and delayed onset of puberty [16]. Additionally, overexpression of *LIN28B* in muscle tissue renders mice under high fat diet resistant to weight gain through increased peripheral glucose uptake and insulin sensitivity [17]. The extent to which *LIN28B* may impact on adiposity and energy metabolism in humans is currently unknown. This study, including 26,636 adult study subjects from the homogenous population of Finland, identified evidence supporting that genetic variation near *LIN28B* impacts body weight, BMI and hip circumference predominantly in females.

In the current study, we chose to genotype two genetic markers located upstream of *LIN28B*, rs7759938 and rs314279. The former has been robustly associated with pubertal timing and adult height. The latter, here associating with female body size and male insulin levels, appears to be less well studied. The pair-wise correlation of rs314279 with rs7759938 (the lead SNP associating with AAM) is only 0.30, and a large meta-analysis including 87,802 women reported evidence also for association between rs314279 and AAM. The finding may indicate the presence of two independent effects at the *LIN28B* region influencing the trait. Nonetheless, this has not been confirmed. Many of the cohorts participating in the meta-analysis study of AAM were lacking data for the marker rs314279, and conditioned analyses interrogating the independent effect of that marker were not carried out [2]. It is possible that rs314279 is poorly covered by large GWA studies in general and that limited data availability might explain why previous GWA studies of BMI, in contrast to our study, have not detected association between BMI and this particular marker [14]. More importantly, in our study the impact of rs314279 on body size appeared stronger when adjusting for the partially correlated rs7759938, but in previous GWA-studies of BMI this model has not been assessed in detail. Finally, whereas our study targets a single, homogenous population, the GWA studies are mostly based on meta-analysis of multiple distinct study cohorts. Therefore, the discrepancy in results might in part originate from a population specific effect, although the accumulated data from association studies of common genetic variants show little evidence for variation in phenotype response between different populations of European descent. Nonetheless, even if our study sample is large, we do acknowledge that further replication studies are needed to verify the findings.

So far only one other study has suggested that genetic variants near *LIN28B* may affect adult BMI [18]. In that study the effect of rs314276 on BMI was evaluated, and the authors found evidence for an age-dependent interaction between the SNP and BMI, in females. Even though rs314276 is completely correlated with one of the markers in the current study, rs7759938, we could not replicate the previous finding. In our study we did not find any evidence for genotype by age interaction at any of the tested marker loci. Of note, the two studies differ by study design, the former being a longitudinal study of a birth cohort and our study representing a cross-sectional study including individuals born between 1923 and 1982. Thus it is possible that varying environmental influences may have prevented detection of an existing age interaction in our study. The disparate results could also be due to differences in the LD-structure between the study populations. Finally, given the power needed to robustly show gene-environment interactions, the results reported in the previous study may represent a false positive effect. Thus, further targeted replication studies are required to elucidate the detailed contribution of *LIN28B* to human body shape and adiposity.

In conclusion, this study shows that genetic variants near *LIN28B* in addition to influencing height growth also associate with female body shape. These data support the accumulating evidence linking *LIN28B-*mediated pathways with adult adiposity-related traits.

## Supporting Information

Figure S1 Height (a and b), weight (c and d), and BMI (e and f) plotted against age in males and females participating in the study. Color intensity reflects the amount of overlapping data points. Blue  =  trend line from linear regression model.
(DOCX)

Table S1 **Descriptive statistics of participating study cohorts.** BMI  =  Body mass index, WHR  =  Waist to hip ratio, ApoA1 =  Apolipoprotein A1, ApoB  =  Apolipoprotein B, FP  =  Fasting Plasma, 2H glucose  =  Plasma glucose concentrations 2 h after a 75 g oral glucose load, FS  =  fasting serum.
(DOCX)

Table S2 **Genotype by sex-interaction analyses of adult anthropometric traits.** Data from the regression analysis of rs7759938 is shown in upper panel and rs314279 in lower panel. The effect allele for both rs7759938 and rs314279 is C. Sex interaction was assessed by a linear regression model including a single marker and sex. BMI  =  body mass index, WHR  =  waist to hip ratio.
(DOCX)

Table S3 **Results from multicollinearity analysis, showing the range of observed variance inflation factors (VIF) for rs7759938/rs314279 in participating cohorts.** The effect allele for both rs7759938 and rs314279 is C. The effect of both SNPs on six anthropometric traits in males, females and both sexes was assessed by a linear regression model including both markers and age in R 2.15.1. Analyses were performed for each cohort separately. The evidence for multicollinearity was assessed by checking the variance inflation factors (VIFs) for the SNPs from these models. Table shows the range of the observed VIFs for each cohort. BMI  =  body mass index, WHR  =  waist to hip ratio. A VIF above 5 was considered indicative of multicollinearity problem.
(DOCX)

Table S4 **Genotype by age-interaction analyses of adult anthropometric traits.** Data from the regression analysis of rs7759938 is shown in upper panel and rs314279 in lower panel. The effect allele for both rs7759938 and rs314279 is C. Age interaction was assessed by a linear regression model including a single marker and age. BMI  =  body mass index, WHR  =  waist to hip ratio.
(DOCX)

Table S5 **Association of genetic variants rs7759938 (upper panel) and rs314279 (lower panel) with adult anthropometric traits by specific age groups.** All phenotypes were standardized prior to analyses. The effect allele for both rs7759938 and rs314279 is C. BMI  =  body mass index, WHR  =  waist to hip ratio.
(DOCX)
