# Supplementary material for: Association of LIN28B with Adult Adiposity-Related Traits in Females
Source: PLoS One. 2012 Nov 13;7(11):e48785. doi: 10.1371/journal.pone.0048785 (PMC3496729; doi:10.1371/journal.pone.0048785)
Supplement: Figure S1 — Height (a and b), weight (c and d), and BMI (e and f) plotted against age in males and females participating in the study. Color intensity reflects the amount of overlapping data points. Blue = trend line from linear regression model. (DOCX) [file pone.0048785.s001.docx]

**Figure S1.** Height (a and b), weight (c and d), and BMI (e and f) plotted against age in males and females participating in the study. Color intensity reflects the amount of overlapping data points. Blue = trend line from linear regression model.


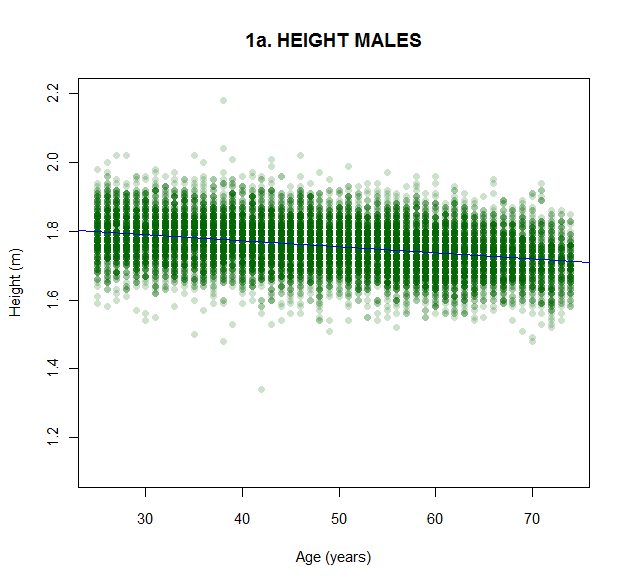


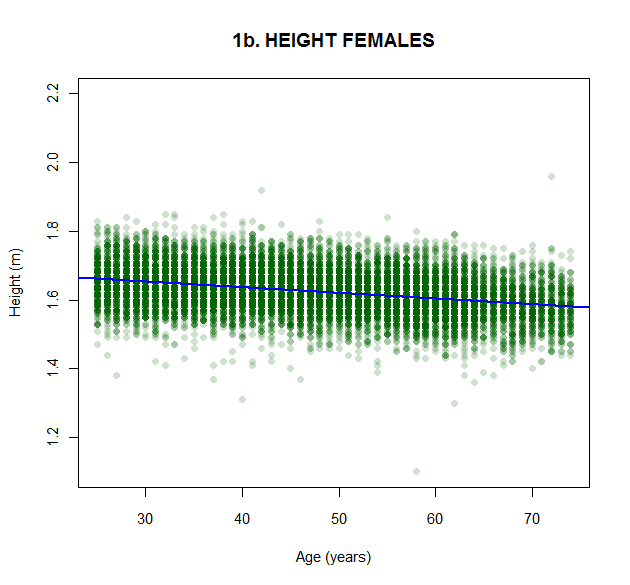


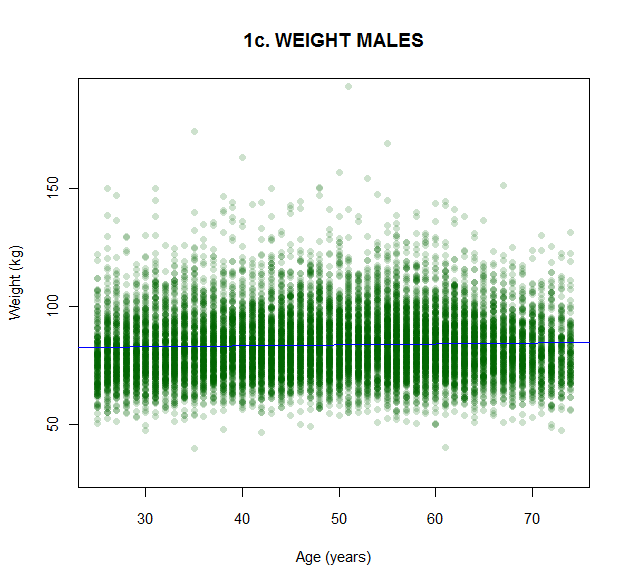


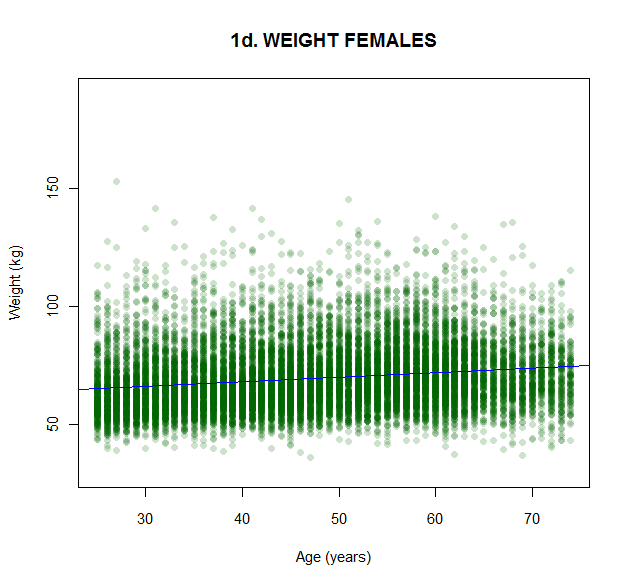


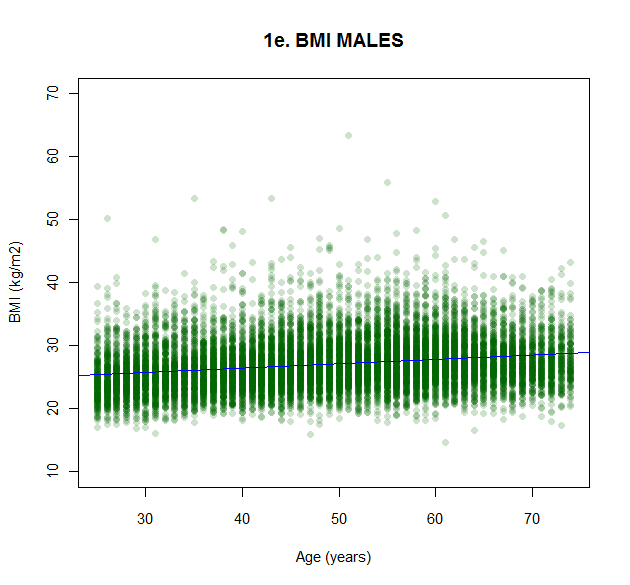


**
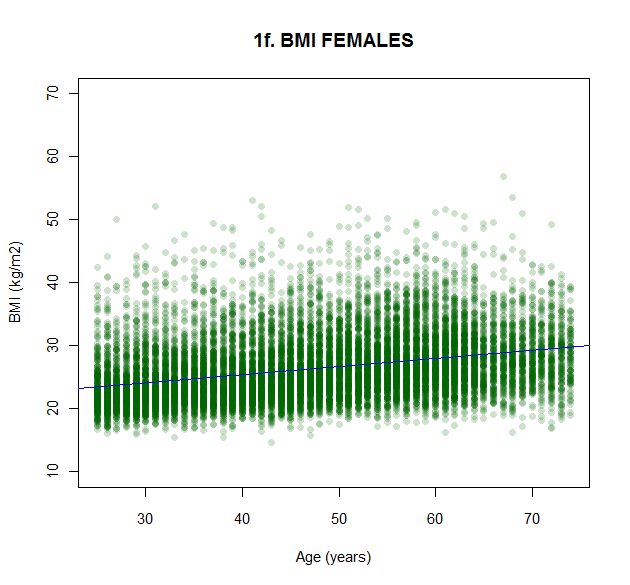
**
