# Supplementary material for: Association of LIN28B with Adult Adiposity-Related Traits in Females
Source: PLoS One. 2012 Nov 13;7(11):e48785. doi: 10.1371/journal.pone.0048785 (PMC3496729; doi:10.1371/journal.pone.0048785)
Supplement: Table S1 — Descriptive statistics of participating study cohorts. BMI = Body mass index, WHR = Waist to hip ratio, ApoA1 = Apolipoprotein A1, ApoB = Apolipoprotein B, FP = Fasting Plasma, 2H glucose = Plasma glucose concentrations 2 h after a 75 g oral glucose load, FS = fasting serum. (DOCX) [file pone.0048785.s002.docx]

**Table S1. Descriptive statistics of participating study cohorts.**

|  |  | |  |  |  | |  |  | |  |
| --- | --- | --- | --- | --- | --- | --- | --- | --- | --- | --- |
| **FINRISK 1992** | **Men** | |  |  | **Women** | |  |  | |  |
| Response Variable | n | Mean (SD) | Min | Max | n | Mean (SD) | | | Min | Max |
| Age(yrs) | 2589 | 44.8 (11.1) | 25 | 64 | 3027 | 44.8 (11.4) | | | 25 | 64 |
| Height(cm) | 2589 | 176.1 (6.8) | 148.0 | 202.0 | 3026 | 162.5 (6.1) | | | 130.0 | 182.0 |
| Weight(kg) | 2589 | 82.5 (13.0) | 46.5 | 154.0 | 3026 | 67.9 (12.6) | | | 36.0 | 137.0 |
| BMI(kg/m2) | 2589 | 26.6 (3.9) | 17.5 | 53.3 | 3025 | 25. 8 (4.9) | | | 15.4 | 52.0 |
| Waist(cm) | 2587 | 94.1 (11.3) | 64.0 | 144.0 | 3027 | 80.4 (11.7) | | | 58.0 | 139.0 |
| Hip(cm) | 2586 | 101.7 (6.6) | 51.0 | 153.0 | 3026 | 101.7 (8.7) | | | 80.5 | 148.0 |
| WHR | 2586 | 0.92 (0.07) | 0.65 | 1.67 | 3025 | 0.79 (0.07) | | | 0.62 | 1.09 |
| Cholesterol (mmol/l) | 1692 | 5.8 (1.1) | 2.4 | 10.5 | 2000 | 5.6 (1.1) | | | 2.4 | 10.3 |
| HDL (mmol/l) | 1693 | 1.3 (0.3) | 0.5 | 2.7 | 2000 | 1.53 (0.34) | | | 0.5 | 2.7 |
| APOA1 (g/l) | 229 | 1.36 (0.23) | 0.38 | 2.04 | 346 | 1.46 (0.24) | | | 0.64 | 2.21 |
| APOB (g/l) | 297 | 1.02 (0.23) | 0.44 | 1.90 | 419 | 0.96 (0.23) | | | 0.49 | 1.80 |
| Triglycerides (mmol/l) | 1693 | 1.9 (1.3) | 0.4 | 12.8 | 2000 | 1.30 (0.87) | | | 0.2 | 14.9 |
|  |  | |  |  |  |  | | |  |  |
| **FINRISK 1997** | **Men** | |  |  | **Women** |  | | |  |  |
| Response Variable | n | Mean (SD) | Min | Max | n | Mean (SD) | | | Min | Max |
| Age(yrs) | 3236 | 48.8 (13.5) | 25 | 74 | 3619 | 47.4 (12.9 | | | 25 | 74 |
| Height(cm) | 3186 | 175.5 (7.1) | 134.0 | 204.0 | 3619 | 162.3 (6.4) | | | 139.0 | 192.0 |
| Weight(kg) | 3185 | 83.0 (13.2) | 40.0 | 150.0 | 3609 | 69.3 (13.3 | | | 37.1 | 137.8 |
| BMI(kg/m2) | 3184 | 27.0 (4.0) | 14.7 | 48.4 | 3608 | 26.4 (5.1) | | | 14.7 | 49.4 |
| Waist(cm) | 3230 | 94.6 (11.4) | 64.5 | 149.0 | 3633 | 81.9 (12.4) | | | 58.0 | 133.5 |
| Hip(cm) | 3228 | 101.9 (6.8) | 77.0 | 146.5 | 3619 | 101.8 (9.2) | | | 69.5 | 159.0 |
| WHR | 3228 | 0.93 (0.07) | 0.68 | 1.20 | 3608 | 0.80 (0.07) | | | 0.61 | 1.38 |
| Cholesterol (mmol/l) | 2085 | 5.6 (1.0) | 2.8 | 10.0 | 2466 | 5.6 (1.1) | | | 2.6 | 9.7 |
| HDL (mmol/l) | 2085 | 1.3 (0.3) | 0.4 | 2.8 | 2466 | 1.5 (0.4) | | | 0.5 | 3.0 |
| APOA1 (g/l) | 1997 | 1.50 (0.26) | 0.35 | 2.61 | 2349 | 1.72 (0.30) | | | 0.48 | 2.97 |
| APOB (g/l) | 1998 | 1.06 (0.25) | 0.21 | 2.09 | 2350 | 0.99 (0.25) | | | 0.30 | 2.04 |
| Triglycerides (mmol/l) | 2085 | 1.7 (1.1) | 0.3 | 11.4 | 2466 | 1.3 (0.8) | | | 0.3 | 11.4 |
|  |  | |  |  |  |  | | |  |  |
| **FINRISK 2002** | **Men** | |  |  | **Women** |  | | |  |  |
| Response Variable | n | Mean (SD) | Min | Max | n | Mean (SD) | | | Min | Max |
| Age(yrs) | 3828 | 48.9 (13.1) | 25 | 74 | 4429 | 47.5 (13.2) | | | 25 | 74 |
| Height(cm) | 3828 | 175.7 (6.8) | 154.0 | 202.0 | 4426 | 162.4 (6.3) | | | 110.0 | 196.0 |
| Weight(kg) | 3828 | 84.3 (14.0) | 48.7 | 174.0 | 4426 | 69.9 (13.5) | | | 38.3 | 145.4 |
| BMI(kg/m2) | 3828 | 27.3 (4.1) | 17.0 | 56.0 | 4425 | 26.5 (5.1) | | | 15.8 | 53.5 |
| Waist(cm) | 3824 | 95.5 (11.8) | 59.0 | 158.0 | 4389 | 83.9 (12.7) | | | 56.5 | 139.0 |
| Hip(cm) | 3827 | 98.6 (7.5) | 76.5 | 141.0 | 4388 | 99.7 (10.1) | | | 73.0 | 149.5 |
| WHR | 3824 | 0.97 (0.07) | 0.59 | 1.25 | 4388 | 0.84 (0.07) | | | 0.63 | 1.34 |
| Cholesterol (mmol/l) | 2510 | 5.8 (1.1) | 2.8 | 10.4 | 3042 | 5.6 (1.0) | | | 3 | 10.5 |
| HDL (mmol/l) | 2509 | 1.4 (0.4) | 0.3 | 3.1 | 3038 | 1.7 (0.4) | | | 0.5 | 3.2 |
| APOA1 (g/l) | 2507 | 1.45 (0.25) | 0.80 | 2.89 | 3033 | 1.63 (0.29) | | | 0.65 | 2.85 |
| APOB (g/l) | 2507 | 1.06 (0.25) | 0.32 | 2.18 | 3033 | 0.96 (0.23 | | | 0.25 | 2.13 |
| Triglycerides (mmol/l) | 2510 | 1.7 (1.1) | 0.3 | 14.2 | 3043 | 1.2 (0.7) | | | 0.3 | 6.9 |
| **FINRISK 2007** | **Men** | |  |  | **Women** |  | | |  |  |
| Response Variable | n | Mean (SD) | Min | Max | n | Mean (SD) | | | Min | Max |
| Age(yrs) | 2753 | 51.2 (13.9) | 25 | 74 | 3155 | 50.2 (14.0) | | | 25 | 74 |
| Height(cm) | 2753 | 176.0 (6.9) | 152.0 | 218.0 | 3155 | 162.8 (6.3) | | | 137.0 | 185.0 |
| Weight(kg) | 2753 | 84.9 (14.2) | 47.5 | 193.3 | 3155 | 71.1 (14.3) | | | 40.1 | 153.0 |
| BMI(kg/m2) | 2753 | 27.4 (4.2) | 16.0 | 63.3 | 3155 | 26.9 (5.4) | | | 16.4 | 53.1 |
| Waist(cm) | 2748 | 98.1 (12.0) | 66.5 | 173.5 | 3106 | 87.9 (13.7) | | | 60.0 | 142.0 |
| Hip(cm) | 2746 | 100.3 (7.7) | 77.0 | 161.0 | 3106 | 101.6 (11.0) | | | 75.5 | 162.0 |
| WHR | 2746 | 0.98 (0.07) | 0.78 | 1.25 | 3105 | 0.86 (0.06) | | | 0.69 | 1.15 |
| Cholesterol (mmol/l) | 1751 | 5.4 (1.0) | 2.2 | 9.4 | 2210 | 5.4 (1.0) | | | 2.6 | 10.0 |
| HDL (mmol/l) | 1750 | 1.3 (0.3) | 0.5 | 3.0 | 2210 | 1.6 (0.4) | | | 0.7 | 3.1 |
| APOA1 (g/l) | 1751 | 1.52 (0.25) | 0.68 | 2.66 | 2210 | 1.70 (0.29) | | | 0.87 | 2.92 |
| APOB (g/l) | 1751 | 0.99 (0.22) | 0.15 | 1.91 | 2210 | 0.91 (0.22) | | | 0.31 | 1.93 |
| Triglycerides (mmol/l) | 1751 | 1.6 (1.0) | 0.3 | 10.8 | 2210 | 1.2 (0.7) | | | 0.3 | 8.4 |
| FP Glucose (mmol/l) | 1853 | 5.88 (0.45) | 4.30 | 7.00 | 2302 | 5.60 (0.46) | | | 4.24 | 6.98 |
| 2H Glucose (mmol/l) | 1841 | 6.12 (1.70) | 2.30 | 11.03 | 2233 | 6.25 (1.64) | | | 2.07 | 11.09 |
| FS Insulin (mU/l) | 1880 | 6.58 (3.41) | 2.40 | 25.90 | 2253 | 6.13 (3.19) | | | 2.40 | 25.60 |

BMI = Body mass index, WHR = Waist to hip ratio, ApoA1 = Apolipoprotein A1, ApoB = Apolipoprotein B, FP = Fasting Plasma, 2H glucose = Plasma glucose concentrations 2 h after a 75g oral glucose load, FS = fasting serum.
