# Supplementary material for: Association of LIN28B with Adult Adiposity-Related Traits in Females
Source: PLoS One. 2012 Nov 13;7(11):e48785. doi: 10.1371/journal.pone.0048785 (PMC3496729; doi:10.1371/journal.pone.0048785)
Supplement: Table S2 — Genotype by sex-interaction analyses of adult anthropometric traits. Data from the regression analysis of rs7759938 is shown in upper panel and rs314279 in lower panel. The effect allele for both rs7759938 and rs314279 is C. Sex interaction was assessed by a linear regression model including a single marker and sex. BMI = body mass index, WHR = waist to hip ratio. (DOCX) [file pone.0048785.s003.docx]

**Table S2. Genotype by sex-interaction analyses of adult anthropometric traits. Data from the regression analysis of rs7759938 is shown in upper panel and rs314279 in lower panel.**

| **rs7759938** |  |  | |  |
| --- | --- | --- | --- | --- |
| **RESPONSE VARIABLE** | **N (M,F)** | **BETA (SE)** | **P** | |
| Height | 26379 (12258, 14121) | 0.014 (0.019) | 0.44 | |
| Weight | 26377 (12257, 14120) | 0.002 (0.019) | 0.90 | |
| BMI | 26375 (12256, 14119) | 0.001 (0.019) | 0.96 | |
| Waist | 26314 (12291, 14023) | -0.015 (0.019) | 0.43 | |
| Hip | 26313 (12289, 14024) | 0.001 (0.019) | 0.95 | |
| WHR | 26307 (12286, 14021) | -0.024 (0.019) | 0.20 | |
|  |  |  |  | |
| **rs314279** |  |  |  | |
| **RESPONSE VARIABLE** | **N (M,F)** | **BETA (SE)** | **P** | |
| Height | 26288 (12213, 14075) | -0.002 (0.026) | 0.95 | |
| Weight | 26286 (12212, 14074) | 0.039 (0.026) | 0.14 | |
| BMI | 26284 (12211, 14073) | 0.042 (0.026) | 0.11 | |
| Waist | 26224 (12246, 13978) | 0.037 (0.026) | 0.16 | |
| Hip | 26223 (12244, 13979) | 0.033 (0.026) | 0.21 | |
| WHR | 26217 (12241, 13976) | 0.031 (0.026) | 0.25 | |

The effect allele for both rs7759938 and rs314279 is C. Sex interaction was assessed by a linear regression model including a single marker and sex. BMI = body mass index, WHR = waist to hip ratio.
