# Supplementary material for: Association of LIN28B with Adult Adiposity-Related Traits in Females
Source: PLoS One. 2012 Nov 13;7(11):e48785. doi: 10.1371/journal.pone.0048785 (PMC3496729; doi:10.1371/journal.pone.0048785)
Supplement: Table S3 — Results from multicollinearity analysis, showing the range of observed variance inflation factors (VIF) for rs7759938/rs314279 in participating cohorts. The effect allele for both rs7759938 and rs314279 is C. The effect of both SNPs on six anthropometric traits in males, females and both sexes was assessed by a linear regression model including both markers and age in R 2.15.1. Analyses were performed for each cohort separately. The evidence for multicollinearity was assessed by checking the variance inflation factors (VIFs) for the SNPs from these models. Table shows the range of the observed VIFs for each cohort. BMI = body mass index, WHR = waist to hip ratio. A VIF above 5 was considered indicative of multicollinearity problem. (DOCX) [file pone.0048785.s004.docx]

**Table S3. Results from multicollinearity analysis, showing the range of observed variance inflation factors (VIF) for rs7759938/rs314279 in participating cohorts.**

| **rs7759938/rs314279** | **FINRISK 1992** | **FINRISK 1997** | **FINRISK 2002** | **FINRISK 2007** |
| --- | --- | --- | --- | --- |
| **RESPONSE VARIABLE** | **VIF** | **VIF** | **VIF** | **VIF** |
| **Height, Weight, BMI, Waist, HIP, WHR** | 1.41-1.42 | 1.45-1.49 | 1.29-1.46 | 1.26-1.50 |

The effect allele for both rs7759938 and rs314279 is C. The effect of both SNPs on six anthropometric traits in males, females and both sexes was assessed by a linear regression model including both markers and age in R 2.15.1. Analyses were performed for each cohort separately. The evidence for multicollinearity was assessed by checking the variance inflation factors (VIFs) for the SNPs from these models. Table shows the range of the observed VIFs for each cohort. BMI = body mass index, WHR = waist to hip ratio. A VIF above 5 was considered indicative of multicollinearity problem.
