# Supplementary material for: Association of LIN28B with Adult Adiposity-Related Traits in Females
Source: PLoS One. 2012 Nov 13;7(11):e48785. doi: 10.1371/journal.pone.0048785 (PMC3496729; doi:10.1371/journal.pone.0048785)
Supplement: Table S4 — Genotype by age-interaction analyses of adult anthropometric traits. Data from the regression analysis of rs7759938 is shown in upper panel and rs314279 in lower panel. The effect allele for both rs7759938 and rs314279 is C. Age interaction was assessed by a linear regression model including a single marker and age. BMI = body mass index, WHR = waist to hip ratio. (DOCX) [file pone.0048785.s005.docx]

**Table S4. Genotype by age-interaction analyses of adult anthropometric traits. Data from the regression analysis of rs7759938 is shown in upper panel and rs314279 in lower panel.**

| **rs7759938** | | | |  | **ALL** |  | | **MALES** |  | **FEMALES** | |  |
| --- | --- | --- | --- | --- | --- | --- | --- | --- | --- | --- | --- | --- |
| **RESPONSE VARIABLE** | **N(M,F)** | | | | **BETA (SE)** | | **P** | **BETA (SE)** | **P** | **BETA (SE)** | **P** | |
| Height | 26379 (12258, 14121) | | | | 0.000 (0.001) | | 0.84 | 0.002 (0.001) | 0.04 | -0.002 (0.001) | 0.09 | |
| Weight | 26377 (12257, 14120) | | | | 0.001 (0.001) | | 0.28 | 0.001 (0.001) | 0.57 | 0.001 (0.001) | 0.34 | |
| BMI | 26375 (12256, 14119) | | | | 0.001 (0.001) | | 0.42 | -0.001 (0.001) | 0.61 | 0.001 (0.001) | 0.12 | |
| Waist | 26314 (12291, 14023) | | | | 0.001 (0.001) | | 0.32 | 0.000 (0.001) | 0.63 | 0.001 (0.001) | 0.37 | |
| Hip | 26313 (12289, 14024) | | | | 0.000 (0.001) | | 0.98 | -0.001 (0.001) | 0.52 | 0.001 (0.001) | 0.53 | |
| WHR | 26307 (12286, 14021) | | | | 0.001 (0.001) | | 0.09 | 0.001 (0.001) | 0.17 | 0.001 (0.001) | 0.31 | |
|  | |  | | |  | |  |  |  |  |  | |
| **rs314279** | | |  | | **ALL** | |  | **MALES** |  | **FEMALES** |  | |
| **RESPONSE**  **VARIABLE** | **N(M,F)** | | | | **BETA (SE)** | | **P** | **BETA (SE)** | **P** | **BETA (SE)** | **P** | |
| Height | 26288 (12213, 14075) | | | | -0.000 (0.001) | | 0.68 | 0.002 (0.001) | 0.27 | -0.002 (0.001) | 0.11 | |
| Weight | 26286 (12212, 14074) | | | | -0.000 (0.001) | | 0.82 | -0.001 (0.001) | 0.57 | 0.000 (0.001) | 0.83 | |
| BMI | 26284 (12211, 14073) | | | | -0.000 (0.001) | | 0.79 | -0.002 (0.001) | 0.20 | 0.001 (0.001) | 0.42 | |
| Waist | 26224 (12246, 13978) | | | | -0.001 (0.001) | | 0.45 | -0.002 (0.001) | 0.23 | 0.000 (0.001) | 0.91 | |
| Hip | 26223 (12244, 13979) | | | | -0.000 (0.001) | | 0.44 | -0.002 (0.001) | 0.20 | 0.000 (0.001) | 0.90 | |
| WHR | 26217 (12241, 13976) | | | | -0.000 (0.001) | | 0.66 | -0.001 (0.001) | 0.51 | 0.000 (0.001) | 0.97 | |

The effect allele for both rs7759938 and rs314279 is C. Age interaction was assessed by a linear regression model including a single marker and age. BMI = body mass index, WHR = waist to hip ratio.
