# Supplementary material for: Association of LIN28B with Adult Adiposity-Related Traits in Females
Source: PLoS One. 2012 Nov 13;7(11):e48785. doi: 10.1371/journal.pone.0048785 (PMC3496729; doi:10.1371/journal.pone.0048785)
Supplement: Table S5 — Association of genetic variants rs7759938 (upper panel) and rs314279 (lower panel) with adult anthropometric traits by specific age groups. All phenotypes were standardized prior to analyses. The effect allele for both rs7759938 and rs314279 is C. BMI = body mass index, WHR = waist to hip ratio. (DOCX) [file pone.0048785.s006.docx]

**Table S5. Association of genetic variants rs7759938 (upper panel) and rs314279 (lower panel) with adult anthropometric traits by specific age groups.**

| **rs7759938** | | |  | |  | **ALL** | |  | | **MALES** | |  | **FEMALES** | |  |  |
| --- | --- | --- | --- | --- | --- | --- | --- | --- | --- | --- | --- | --- | --- | --- | --- | --- |
| **AGE-GROUP** | **RESPONSE**  **VARIABLE** | | | **N(M,F)** | | **BETA (SE)** | **P** | | **BETA (SE)** | | **P** | | **BETA (SE)** | **P** | | |
| 25-34yrs | Height | | | 5191 (2292, 2899) | | **0.058 (0.020)** | **0.004** | | 0.010 (0.031) | | 0.75 | | **0.094 (0.027)** | **0.0005** | | |
| 35-44yrs | Height | | | 5857 (2669, 3188) | | 0.034 (0.019) | 0.06 | | 0.019 (0.028) | | 0.50 | | 0.046 (0.025) | 0.06 | | |
| 45-54yrs | Height | | | 6033 (2759, 3274) | | 0.014 (0.018) | 0.43 | | -0.006 (0.027) | | 0.82 | | 0.031 (0.024) | 0.20 | | |
| 55-64yrs | Height | | | 6296 (2974, 3322) | | **0.050 (0.018)** | **0.005** | | 0.048 (0.026) | | 0.06 | | 0.051 (0.024) | 0.04 | | |
| 65-74yrs | Height | | | 3002 (1564, 1438) | | 0.051 (0.026) | 0.05 | | **0.101 (0.036)** | | **0.004** | | -0.007 (0.038) | 0.85 | | |
| 25-34yrs | Weight | | | 5191 (2292, 2899) | | 0.019 (0.020) | 0.35 | | 0.018 (0.032) | | 0.56 | | 0.020 (0.027) | 0.46 | | |
| 35-44yrs | Weight | | | 5857 (2669, 3188) | | 0.010 (0.020) | 0.63 | | 0.027 (0.030) | | 0.36 | | -0.004 (0.026) | 0.87 | | |
| 45-54yrs | Weight | | | 6034 (2759, 3275) | | 0.007 (0.020) | 0.73 | | -0.010 (0.029) | | 0.74 | | 0.020 (0.027) | 0.44 | | |
| 55-64yrs | Weight | | | 6294 (2973, 3321) | | 0.018 (0.019) | 0.34 | | 0.013 (0.028) | | 0.65 | | 0.023 (0.026) | 0.38 | | |
| 65-74yrs | Weight | | | 3001 (1564, 1437) | | 0.039 (0.026) | 0.13 | | 0.039 (0.035) | | 0.26 | | 0.040 (0.038) | 0.30 | | |
| 25-34yrs | BMI | | | 5191 (2292, 2899) | | -0.002 (0.019) | 0.92 | | 0.015 (0.029) | | 0.60 | | -0.014 (0.025) | 0.57 | | |
| 35-44yrs | BMI | | | 5857 (2669, 3188) | | -0.004 (0.019) | 0.83 | | 0.017 (0.029) | | 0.55 | | -0.020 (0.025) | 0.42 | | |
| 45-54yrs | BMI | | | 6033 (2759, 3274) | | 0.000 (0.019) | 0.98 | | -0.010 (0.029) | | 0.74 | | 0.009 (0.026) | 0.74 | | |
| 55-64yrs | BMI | | | 6294 (2973, 3321) | | -0.007 (0.019) | 0.70 | | -0.017 (0.027) | | 0.54 | | 0.001 (0.026) | 0.97 | | |
| 65-74yrs | BMI | | | 3000 (1563, 1437) | | 0.009 (0.026) | 0.72 | | -0.016 (0.036) | | 0.66 | | 0.038 (0.038) | 0.32 | | |
| 25-34yrs | Waist | | | 5123 (2293, 2830) | | 0.007 (0.019) | 0.70 | | 0.016 (0.029) | | 0.57 | | 0.000 (0.025) | 0.99 | | |
| 35-44yrs | Waist | | | 5824 (2668, 3156) | | 0.018 (0.018) | 0.33 | | 0.038 (0.027) | | 0.17 | | 0.001 (0.024) | 0.95 | | |
| 45-54yrs | Waist | | | 6029 (2758, 3271) | | 0.012 (0.019) | 0.53 | | 0.015 (0.028) | | 0.60 | | 0.010 (0.026) | 0.71 | | |
| 55-64yrs | Waist | | | 6286 (2967, 3319) | | 0.012 (0.019) | 0.51 | | 0.021 (0.027) | | 0.44 | | 0.004 (0.026) | 0.86 | | |
| 65-74yrs | Waist | | | 3052 (1605, 1447) | | 0.030 (0.026) | 0.25 | | 0.026 (0.035) | | 0.45 | | 0.033 (0.038) | 0.38 | | |
| 25-34yrs | Hip | | | 5124 (2293, 2831) | | 0.024 (0.020) | 0.23 | | 0.043 (0.031) | | 0.17 | | 0.011 (0.026) | 0.67 | | |
| 35-44yrs | Hip | | | 5825 (2668, 3157) | | 0.009 (0.019) | 0.63 | | 0.022 (0.028) | | 0.43 | | -0.002 (0.026) | 0.95 | | |
| 45-54yrs | Hip | | | 6028 (2757, 3271) | | -0.007 (0.020) | 0.72 | | -0.012 (0.028) | | 0.66 | | -0.002 (0.026) | 0.93 | | |
| 55-64yrs | Hip | | | 6286 (2967, 3319) | | 0.007 (0.019) | 0.71 | | -0.007 (0.027) | | 0.80 | | 0.019 (0.026) | 0.46 | | |
| 65-74yrs | Hip | | | 3050 (1604, 1446) | | 0.019 (0.027) | 0.47 | | 0.015 (0.038) | | 0.69 | | 0.024 (0.038) | 0.53 | | |
| 25-34yrs | WHR | | | 5123 (2293, 2830) | | -0.017 (0.019) | 0.35 | | -0.013 (0.028) | | 0.64 | | -0.021 (0.025) | 0.40 | | |
| 35-44yrs | WHR | | | 5823 (2667, 3156) | | 0.022 (0.018) | 0.20 | | 0.043 (0.027) | | 0.11 | | 0.006 (0.024) | 0.79 | | |
| 45-54yrs | WHR | | | 6028 (2757, 3271) | | 0.030 (0.019) | 0.11 | | 0.037 (0.027) | | 0.17 | | 0.024 (0.026) | 0.36 | | |
| 55-64yrs | WHR | | | 6285 (2966, 3319) | | 0.012 (0.018) | 0.52 | | 0.035 (0.025) | | 0.16 | | -0.013 (0.026) | 0.60 | | |
| 65-74yrs | WHR | | | 3048 (1603, 1445) | | 0.027 (0.026) | 0.29 | | 0.028 (0.033) | | 0.40 | | 0.026 (0.041) | 0.53 | | |
|  |  | | |  | |  |  | |  | |  | |  |  | | |
| **rs314279** | |  | |  | | **ALL** |  | | **MALES** | |  | | **FEMALES** |  | | |
| **AGE-GROUP** | **RESPONSE**  **VARIABLE** | | | **N(M,F)** | | **BETA (SE)** | **P** | | **BETA (SE)** | | **P** | | **BETA (SE)** | **P** | | |
| 25-34yrs | Height | | | 5174 (2289, 2885) | | 0.024 (0.029) | 0.39 | | -0.009 (0.043) | | 0.83 | | 0.052 (0.038) | 0.18 | | |
| 35-44yrs | Height | | | 5834 (2656, 3178) | | 0.071 (0.027) | 0.008 | | 0.046 (0.040) | | 0.25 | | 0.091 (0.036) | 0.01 | | |
| 45-54yrs | Height | | | 6010 (2752, 3258) | | 0.039 (0.025) | 0.13 | | 0.036 (0.037) | | 0.34 | | 0.040 (0.035) | 0.24 | | |
| 55-64yrs | Height | | | 6267 (2952, 3315) | | 0.016 (0.025) | 0.51 | | -0.001 (0.036) | | 0.97 | | 0.033 (0.034) | 0.34 | | |
| 65-74yrs | Height | | | 3003 (1564, 1439) | | 0.046 (0.037) | 0.21 | | 0.108 (0.051) | | 0.04 | | -0.020 (0.053) | 0.71 | | |
| 25-34yrs | Weight | | | 5174 (2289, 2885) | | 0.040 (0.029) | 0.16 | | 0.008 (0.043) | | 0.85 | | 0.064 (0.038) | 0.09 | | |
| 35-44yrs | Weight | | | 5834 (2656, 3178) | | 0.066 (0.028) | 0.02 | | **0.119 (0.042)** | | **0.004** | | 0.022 (0.038) | 0.56 | | |
| 45-54yrs | Weight | | | 6011 (2752, 3259) | | 0.047 (0.028) | 0.10 | | -0.011 (0.041) | | 0.80 | | 0.095 (0.038) | 0.01 | | |
| 55-64yrs | Weight | | | 6265 (2951, 3314) | | 0.029 (0.027) | 0.27 | | -0.004 (0.039) | | 0.91 | | 0.059 (0.037) | 0.11 | | |
| 65-74yrs | Weight | | | 3002 (1564, 1438) | | 0.024 (0.037) | 0.51 | | 0.022 (0.050) | | 0.66 | | 0.027 (0.053) | 0.61 | | |
| 25-34yrs | BMI | | | 5174 (2289, 2885) | | 0.036 (0.026) | 0.18 | | 0.019 (0.040) | | 0.64 | | 0.048 (0.035) | 0.17 | | |
| 35-44yrs | BMI | | | 5834 (2656, 3178) | | 0.039 (0.027) | 0.14 | | 0.103 (0.040) | | 0.01 | | -0.013 (0.036) | 0.73 | | |
| 45-54yrs | BMI | | | 6010 (2752, 3258) | | 0.026 (0.027) | 0.33 | | -0.036 (0.041) | | 0.38 | | 0.078 (0.037) | 0.04 | | |
| 55-64yrs | BMI | | | 6265 (2951, 3314) | | 0.020 (0.026) | 0.46 | | -0.009 (0.038) | | 0.82 | | 0.045 (0.036) | 0.22 | | |
| 65-74yrs | BMI | | | 3001 (1563, 1438) | | -0.002 (0.037) | 0.96 | | -0.037 (0.052) | | 0.48 | | 0.036 (0.054) | 0.51 | | |
| 25-34yrs | Waist | | | 5107 (2290, 2817) | | 0.033 (0.026) | 0.21 | | 0.026 (0.040) | | 0.51 | | 0.038 (0.035) | 0.27 | | |
| 35-44yrs | Waist | | | 5891 (2655, 3146) | | 0.040 (0.026) | 0.12 | | 0.095 (0.038) | | 0.01 | | -0.006 (0.036) | 0.87 | | |
| 45-54yrs | Waist | | | 6006 (2751, 3255) | | 0.026 (0.027) | 0.33 | | -0.025 (0.039) | | 0.53 | | 0.071 (0.037) | 0.05 | | |
| 55-64yrs | Waist | | | 6257 (2945, 3312) | | 0.011 (0.026) | 0.68 | | -0.021 (0.037) | | 0.58 | | 0.039 (0.036) | 0.27 | | |
| 65-74yrs | Waist | | | 3053 (1605, 1448) | | -0.011 (0.037) | 0.76 | | -0.015 (0.050) | | 0.77 | | -0.007 (0.054) | 0.89 | | |
| 25-34yrs | Hip | | | 5108 (2290, 2818) | | 0.055 (0.028) | 0.05 | | 0.052 (0.043) | | 0.23 | | 0.057 (0.037) | 0.13 | | |
| 35-44yrs | Hip | | | 5802 (2655, 3147) | | 0.059 (0.027) | 0.03 | | 0.106 (0.039) | | 0.008 | | 0.018 (0.037) | 0.63 | | |
| 45-54yrs | Hip | | | 6005 (2750, 3255) | | 0.032 (0.027) | 0.24 | | -0.021 (0.040) | | 0.60 | | 0.078 (0.037) | 0.04 | | |
| 55-64yrs | Hip | | | 6257 (2945, 3312) | | 0.008 (0.026) | 0.78 | | -0.026 (0.038) | | 0.50 | | 0.037 (0.036) | 0.30 | | |
| 65-74yrs | Hip | | | 3051 (1604, 1447) | | 0.045 (0.038) | 0.24 | | 0.041 (0.054) | | 0.44 | | 0.048 (0.053) | 0.37 | | |
| 25-34yrs | WHR | | | 5107 (2290, 2817) | | -0.001 (0.026) | 0.97 | | -0.007 (0.038) | | 0.87 | | 0.004 (0.036) | 0.91 | | |
| 35-44yrs | WHR | | | 5800 (2654, 3146) | | 0.012 (0.025) | 0.64 | | 0.060 (0.037) | | 0.11 | | -0.028 (0.034) | 0.41 | | |
| 45-54yrs | WHR | | | 6005 (2750, 3255) | | 0.010 (0.026) | 0.71 | | -0.020 (0.038) | | 0.60 | | 0.038 (0.037) | 0.31 | | |
| 55-64yrs | WHR | | | 6256 (2944, 3312) | | 0.009 (0.025) | 0.71 | | -0.014 (0.035) | | 0.70 | | 0.034 (0.036) | 0.36 | | |
| 65-74yrs | WHR | | | 3049 (1603, 1446) | | -0.061 (0.037) | 0.10 | | -0.061 (0.048) | | 0.20 | | -0.061 (0.057) | 0.29 | | |

All phenotypes were standardized prior to analyses. The effect allele for both rs7759938 and rs314279 is C. BMI = body mass index, WHR = waist to hip ratio.
